# Monoclonal Antibodies Targeting Surface-Exposed Epitopes of Candida albicans Cell Wall Proteins Confer *In Vivo* Protection in an Infection Model

**DOI:** 10.1128/aac.01957-21

**Published:** 2022-03-14

**Authors:** Soumya Palliyil, Mark Mawer, Sami A. Alawfi, Lily Fogg, Tyng H. Tan, Giuseppe Buda De Cesare, Louise A. Walker, Donna M. MacCallum, Andrew J. Porter, Carol A. Munro

**Affiliations:** a Scottish Biologics Facility, Institute of Medical Sciences, School of Medicine, Medical Sciences and Nutrition, University of Aberdeen, Aberdeen, United Kingdom; b Aberdeen Fungal Group, Institute of Medical Sciences, School of Medicine, Medical Sciences and Nutrition, University of Aberdeen, Aberdeen, United Kingdom

**Keywords:** *Candida albicans*, anti-*Candida* mAbs, immunotherapy, monoclonal antibodies

## Abstract

Monoclonal antibody (mAb)-based immunotherapies targeting systemic and deep-seated fungal infections are still in their early stages of development, with no licensed antifungal mAbs currently being available for patients at risk. The cell wall glycoproteins of Candida albicans are of particular interest as potential targets for therapeutic antibody generation due to their extracellular location and key involvement in fungal pathogenesis. Here, we describe the generation of recombinant human antibodies specifically targeting two key cell wall proteins (CWPs) in C. albicans: Utr2 and Pga31. These antibodies were isolated from a phage display antibody library using peptide antigens representing the surface-exposed regions of CWPs expressed at elevated levels during *in vivo* infection. Reformatted human-mouse chimeric mAbs preferentially recognized C. albicans hyphal forms compared to yeast cells, and increased binding was observed when the cells were grown in the presence of the antifungal agent caspofungin. In J774.1 macrophage interaction assays, mAb pretreatment resulted in the faster engulfment of C. albicans cells, suggesting a role of the CWP antibodies as opsonizing agents during phagocyte recruitment. Finally, in a series of clinically predictive mouse models of systemic candidiasis, our lead mAb achieved improved survival (83%) and a several-log reduction of the fungal burden in the kidneys, similar to the levels achieved for the fungicidal drug caspofungin and superior to the therapeutic efficacy of any anti-*Candida* mAb reported to date.

## INTRODUCTION

Invasive fungal infections (IFIs) are serious, life-threatening conditions typically affecting individuals with a compromised immune system, including patients with hematological malignancies and those undergoing cytotoxic chemotherapy and organ transplantation (1). Ironically, due to advancements in immunomodulatory drugs (including antibodies), patients suffering from cancer and other complex health conditions are often left with a temporary but weakened immune system due to their medication. This drug-induced immune suppression has caused a significant rise in life-threatening systemic and organ-specific fungal infections (1). Clinicians suffer the frustration of successfully controlling cancers only to see their patients succumb to these often difficult-to-treat infections. Opportunistic pathogenic fungi, including Aspergillus, *Candida*, and Cryptococcus species, are responsible for invasive fungal infections and the death of at least 1.7 million people each year globally (2). Population-based surveillance studies show that the yearly incidence of invasive Candida albicans infections and those caused by related species, including Candida parapsilosis, Candida glabrata, and Candida tropicalis, can be as high as 21 per 100,000 in some geographical locations (1). Furthermore, the ongoing global coronavirus disease 2019 (COVID-19) pandemic has fueled an increase in secondary fungal superinfections, such as severe acute respiratory syndrome coronavirus 2 (SARS-CoV-2)-associated pulmonary aspergillosis (CAPA), with mortality rates of >40% in almost all study cohorts (3).

Currently, IFIs are treated with antifungal agents belonging to four main drug classes and include amphotericin B, fluconazole, voriconazole, caspofungin, and 5-flucytosine. The recalcitrance of some infections has encouraged longer-term treatment regimens and even their prophylactic use for some surgeries and organ transplantations. This unmanaged use of a limited drug armory has inevitably led to the emergence of antifungal drug resistance in many fungal genera (4, 5). Certain *Candida* species, such as Candida auris, are of particular concern in several countries as some isolates show reduced susceptibility to fluconazole, amphotericin B, and echinocandins, with clinicians labeling C. auris the methicillin-resistant Staphylococcus aureus (MRSA) of the fungal world (6, 7). The increasingly well-documented shortcomings of our existing antifungals (toxicity, complex drug-drug interactions, and the emergence of multidrug-resistant strains) and the intrinsic ability of certain fungal species to evade drug therapies have accelerated the need to develop novel “first-in-class” alternatives to tackle these life-threatening conditions.

In all pathogenic fungi of consequence, the cell wall is a dynamic structure continuously changing in response to body/culture conditions and environmental stimuli. The cell wall of C. albicans is covered in an outer layer of glycoproteins that play important roles in pathogenesis and mediating interactions between the host and the fungus (8). These proteins “immune-mask” fungal β-glucans from recognition by the mammalian β-glucan receptor dectin-1 (9). Some of these cell surface glycoproteins, including adhesins, invasins, and superoxide dismutases, are also important virulence factors in their own right. Often shed by the invading fungus, these proteins promote the adherence of C. albicans to host cells, mediate tissue invasion, and combat oxidative burst defenses (10). Many cell surface glycoproteins are posttranslationally modified by the addition of a glycosylphosphatidylinositol (GPI) anchor and can be either fungal plasma membrane localized or translocated into the cell wall, where they are covalently attached to the β-(1,6)-glucan polymer (11). More than 100 putative GPI-anchored proteins have been annotated in C. albicans using *in silico* analyses (12, 13), with some having enzymatic functions associated with cell wall biosynthesis and cell wall remodeling. Several studies have reported alterations in cell wall protein (CWP) composition and expression in response to changes in growth conditions, including carbon source, iron limitation, hypoxia, or antifungal drug challenge, indicating the possibility of the up/downregulation of these proteins during *in vivo* infection (14).

While CWPs define the success of many fungal pathogens, some may also provide an “Achilles’ heel” that can be exploited in therapy. Neutralizing antibodies against CWPs have been detected in patients’ sera and therefore represent an important source of antigens/epitopes for vaccine generation and therapeutic antibody development (15). GPI-anchored CWPs, such as Pga31 and Utr2, play important roles in cell wall integrity and assembly (16). Utr2 carries a glycoside hydrolase family 16 domain, predictive of transglycosidase activity that catalyzes cross-links between β-(1,3)-glucan and chitin, as shown for the Saccharomyces cerevisiae orthologue Crh1, and is involved in cell wall remodeling and maintenance (17). In C. albicans, *UTR2*, *CRH11*, and *CRH12* belong to the *CRH* gene family and are strongly regulated by calcineurin, a serine/threonine protein phosphatase involved in cell wall morphogenesis and virulence (18, 19). Mutants lacking *UTR2* exhibit defective cell wall organization (inducing the cell integrity mitogen-activated protein [MAP] kinase signaling pathway), reductions in adherence to mammalian cells, and reduced virulence, resulting in the prolonged survival of animals in *in vivo* models of systemic infection (18, 20). Immunofluorescence staining locates Utr2 predominantly to the budding site of mother yeast cells, eventually forming a ring at the base of the neck, whereas during hyphal elongation, the protein is detected at the tip of the germ tube and as a ring at the septum (18). Utr2 colocalizes to chitin-rich regions in yeast, pseudohyphal, and hyphal forms (18).

Another GPI-anchored glycoprotein, Pga31, which has an unknown function, is upregulated in the opaque form of C. albicans and after exposure to caspofungin (12, 21). A *pga31*-null mutant exhibits decreased chitin content compared to the wild-type (wt) strain and increased sensitivity to caspofungin and cell wall-perturbing agents such as calcofluor white (CFW) and SDS (12). The low-chitin phenotype of the *pga31*-null mutant points to a role for Pga31 linked to chitin assembly during cell wall biogenesis and the maintenance of wall integrity under cellular stress.

Given their established roles in cell wall remodeling, upregulation after caspofungin treatment (22), and enhanced expression in *in vivo* models of systemic candidiasis (C. A. Munro, unpublished data), we investigated the potential of Utr2 and Pga31 as therapeutic targets for the development of monoclonal antibodies (mAbs) to treat life-threatening fungal infections. mAb-based therapies have seen unprecedented levels of success in cancer and autoimmune disorders, producing several blockbuster drugs, including adalimumab (Humira). This molecule class has also expanded into novel therapeutic modalities such as bispecific antibodies and antibody-drug conjugates (ADCs) (23, 24).

Cancer treatments were once dominated by toxic small-molecule therapies requiring a balancing act between killing the cancer cells and damaging healthy cells. However, cancer therapy was revolutionized by targeted mAb therapies that have increased efficacy and reduced side effects. This balancing act is also practiced by infectious disease clinicians treating life-threatening systemic fungal infections where toxic molecules are used to kill the fungus without “killing” the patient. Unfortunately, mAb technology has failed to generate a significant impact on the infectious disease field to date, but the increasing emergence of drug-resistant fungal strains is accelerating the need for new therapeutic modalities. Currently, only a few antifungal mAbs have been reported to show modest efficacy against *in vivo* infection, and none of these have so far progressed into the clinic (25–27).

In this study, the C. albicans cell wall proteome was interrogated using trypsin digestion followed by liquid chromatography-tandem mass spectrometry (LC-MS/MS) analysis to identify several covalently linked CWPs, including Utr2 and Pga31, and their surface-exposed epitopes. These epitopes were used to generate monoclonal antibodies from a naive human phage display antibody library. The ability of these recombinant mAbs to bind several pathogenic fungi was investigated, with some showing fungus-specific and others showing fungal species-specific binding. However, most importantly, their protective efficacy has been demonstrated in a murine model of systemic candidiasis, with potency approaching that of more traditional antifungal drug classes.

## RESULTS

### Antigen design for recombinant antibody generation.

Guided by the cell wall proteome analysis of various caspofungin-susceptible and -resistant strains of C. albicans, peptide sequences accessible to trypsin digestion were identified and matched with their respective fungal cell wall proteins (Tables 1 and 2) (22). Based on the predicted β turn structures (NetTurnP 1.0 algorithm) and hydropathy of these surface-exposed regions, peptide sequences were selected as antigens representing the CWPs Utr2 and Pga31. β turn regions are often solvent-exposed secondary structures and tend to have a relatively higher propensity for antibody binding (28). A small panel of trypsin-susceptible peptides from both proteins was custom synthesized and C-terminally biotinylated via an additionally introduced lysine residue, and these conjugates were used as antigens for biopanning experiments.

**TABLE 1:** Amino acid sequences of the tryptically digested peptides identified by cell wall proteome analysis of C. albicans SC5314 using the LC-MS/MS method

Utr2p peptide detected by LC-MS/MS	Pga31p peptide detected by LC-MS/MS
MSTFQESFDSK	HEGAALNYLFLAAPGVAENLK
IQFSLWPGGDSSNAK	QPLNVGNTVLQLGGSGDGTK
YGYYYAHIK	VDIAEDGTLSFDGSDSVGAAK
EIYATAYDIPNDVK	NINDPYNYSK
GTIEWAGGLINWDSEDIKK	

**TABLE 2:** Mapping of the tryptic peptides identified by cell wall proteome analysis onto the C. albicans Utr2p and Pga31p sequences^*a*^

Protein	Associated protein sequence determined by the *Candida* Genome Database^*b*^
Utr2p	MRFSTLHFAFLATLSSIFTVVAASDTTTCSSSKHCPEDKPCCSQFGICGTGAYCLGGCDIRYSYNLTACMPMPR**MSTFQESFDSK**DKVKEIELQSDYLGNSTEADWVYTGWVDYYDNSLLIQMPNHTTGTVVSSTKYLWYGKVGATLKTSHDGGVVTAFILFSDVQDEIDYEFVGYNLTNPQSNYYSQGILNYNNSRNSSVNNTFEYYHNYEMDWTEDKIEWYIDGEKVRTLNKNDTWNETSNRYDYPQTPSR**IQFSLWPGGDSSNAKGTIEWAGGLINWDSEDIKKYGYYYAHIKEIYATAYDIPNDVK**LDGNSTKESDYHAFLYNSTDGDASNIMLTTKKTWLGSDDATGFDPQNDDEDSSSNKAQETTITSVSGSSTITSVKTDSTKKTANVPAQNTAAAAQATAKSSTGTNTYDPSAGVGGFVQDSKSTDSGSSGSSSQGVANSLNESVISGIFASICLGILSFFM*
Pga31p	MKFHMRLQKKIFVLEYYIKPDISSFSGKYLFLLFFLFQSHINQLFDYIYFIQKYLICYIMKFLTAASLLTLSSSALAAIKDIQLYAQSSNNEVNDFGISSR**HEGAALNYLFLAAPGVAENLK**YDDETKTVYTELKAGSSTVR**QPLNVGNTVLQLGGSGDGTKVDIAEDGTLSFDGSDSVGAAKNINDPYNYS**KDSYAVVKGGDGAIPIKLVAKFTGDDKESASSSSSSAAPEPTASSSEAPKETPVYSNSTVTLYTTYCPLSTTITLTVCSDVCTPTVIETSGSVTVSSVQVPSKTASSEAAPPKTTVDSVSKPAPSGKKPTAAVTSFEGAANALTGGSVAIAVAAAIGLVF*

a Peptides are shown in boldface type within the amino acid sequences of each protein. The chitin binding region of Utr2, identified using SMART tool analysis software (http://smart.embl-heidelberg.de), is underlined. * denotes translation termination codon.

b See http://www.candidagenome.org/cgi-bin/protein/proteinPage.pl?dbid=CAL0000104 and http://www.candidagenome.org/cgi-bin/protein/proteinPage.pl?dbid=CAL0004244.

### Isolation of C. albicans CWP-specific recombinant antibody fragments from a human antibody library through biopanning.

Phage display technology was employed to isolate Utr2 and Pga31 peptide-specific antibody fragments from a human antibody library in single-chain fragment-variable (scFv) format. Following three rounds of selection using decreasing concentrations of biotinylated peptide antigen, 2 phage clones specific for the Pga31 peptide and 14 positive binders that recognized the Utr2 peptide were isolated, and their unique scFv genes were confirmed by DNA sequencing. These clones were reformatted into soluble single-chain antibodies (scAbs) by cloning their respective VH-linker-VL regions into the bacterial expression vector pIMS147 (29) to facilitate detection in biochemical assays and quantification of soluble protein expressed by Escherichia coli TG1 cells.

**FIG 1:**
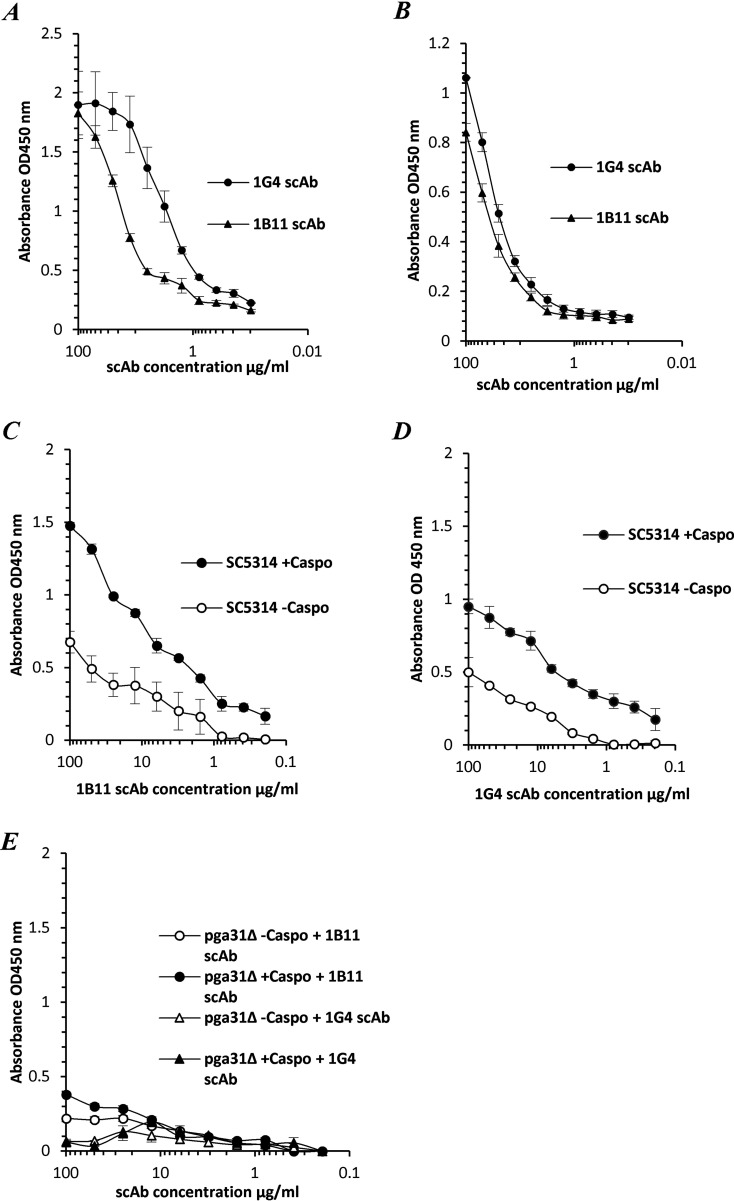
Pga31 scAb binding profiles. (A) Antigen binding ELISA where wells are coated with a Pga31 peptide-biotin conjugate. (B) scAb binding to C. albicans SC5314 hyphae. (C) scAb 1B11 binding to total cell lysates of C. albicans SC5314 treated with or without 0.032 μg/mL caspofungin (Caspo). (D) scAb 1G4 binding to total cell lysates of C. albicans SC5314 treated with or without 0.032 μg/mL caspofungin. (E) Lack of binding of the 1B11 and 1G4 scAbs to the cell lysates of the C. albicans *pga31*Δ mutant strain treated with or without 0.032 μg/mL caspofungin. Doubling dilutions of scAbs were added to the plates coated with the Pga31 peptide conjugate and C. albicans SC5314 (with or without caspofungin) or *pga31*Δ mutant (with or without caspofungin) cell wall lysates and detected using an anti-human C kappa HRP-conjugated secondary antibody. Values represent the mean absorbance (OD_450_) readings (*n* = 2 [samples run in duplicate]), and error bars denote the standard errors of the means (SEM).

scAbs from Pga31 biopanning reacted specifically to their peptide antigen and C. albicans wild-type strain SC5314 hyphae (Fig. 1A and B) and bound total cell lysates of C. albicans SC5314 treated with or without 0.032 μg/mL caspofungin (Fig. 1C and D). An increase in the scAb binding signal was recorded when the lysate was prepared from the cells treated with caspofungin (Fig. 1C and D). This reinforced the hypothesis that Pga31 is overexpressed when the cells are grown in caspofungin and that it is involved in cell wall integrity (22). As a confirmatory control experiment, the scAbs did not bind to a *pga31*Δ mutant strain treated with/without caspofungin (Fig. 1E).

**FIG 2:**
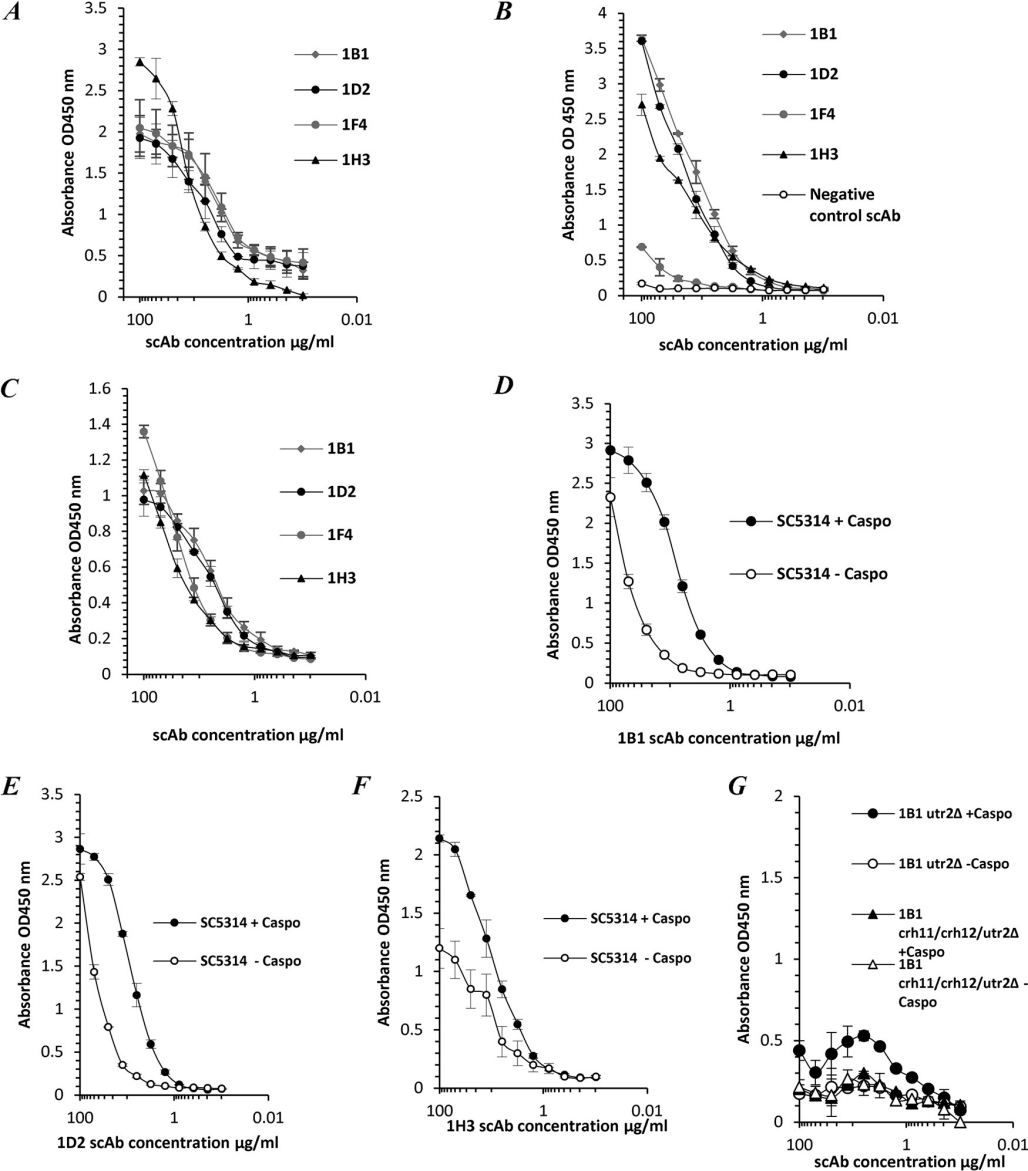
Utr2 scAb binding profiles. Utr2 scAb binding activities specific for the immobilized Utr2 peptide (A); scAb binding using a cell lysate preparation of C. albicans SC5314 (B); scAb binding against C. albicans SC5314 hyphae (C); and scAb 1B1, 1D2, and 1H3 binding to C. albicans SC5314 yeast cells treated with or without 0.032 μg/mL caspofungin (D to F). (G) Representative scAb 1B1 binding to the cell lysates of a *utr2* single mutant (*utr2*Δ) and a triple mutant (*utr2*Δ/*crh11*Δ/*crh12*Δ) treated with or without caspofungin at 0.032 μg/mL. scAbs 1H3, 1D2, and 1F4 also showed similar binding profiles with *utr2*Δ mutant strains (results not shown). Values represent mean absorbance (OD_450_) readings (*n* = 2 [samples run in duplicate]). Error bars denote the SEM.

The antigen binding of the top 4 Utr2-specific scAbs, 1B1, 1D2, 1F4, and 1H3, selected based on peptide-biotin conjugate enzyme-linked immunosorbent assay (ELISA) signals, was confirmed (Fig. 2A), and these clones were tested for their ability to recognize Utr2 in cell lysate preparations of C. albicans SC5314 yeast cells (Fig. 2B). A nonspecific negative-control scAb (human scFv against an unrelated antigen) was unable to bind to the C. albicans cell lysate (Fig. 2B). The surface exposure and epitope accessibility of the Utr2 antigen peptide were established, with all scAbs binding to C. albicans hyphal cells (Fig. 2C). When yeast cells were treated with 0.032 μg/mL caspofungin, an increase in scAb binding was observed compared to fungal cells grown in the absence of the drug (Fig. 2D to F). No scAb binding was seen when the C. albicans single mutant strain *utr2*Δ and triple mutant strain *utr2*Δ/*crh11*Δ/*crh12*Δ were used (Fig. 2G).

### Reformatting scAbs into human-mouse chimeric IgGs for *in vitro* and *in vivo* validation studies.

**FIG 3:**
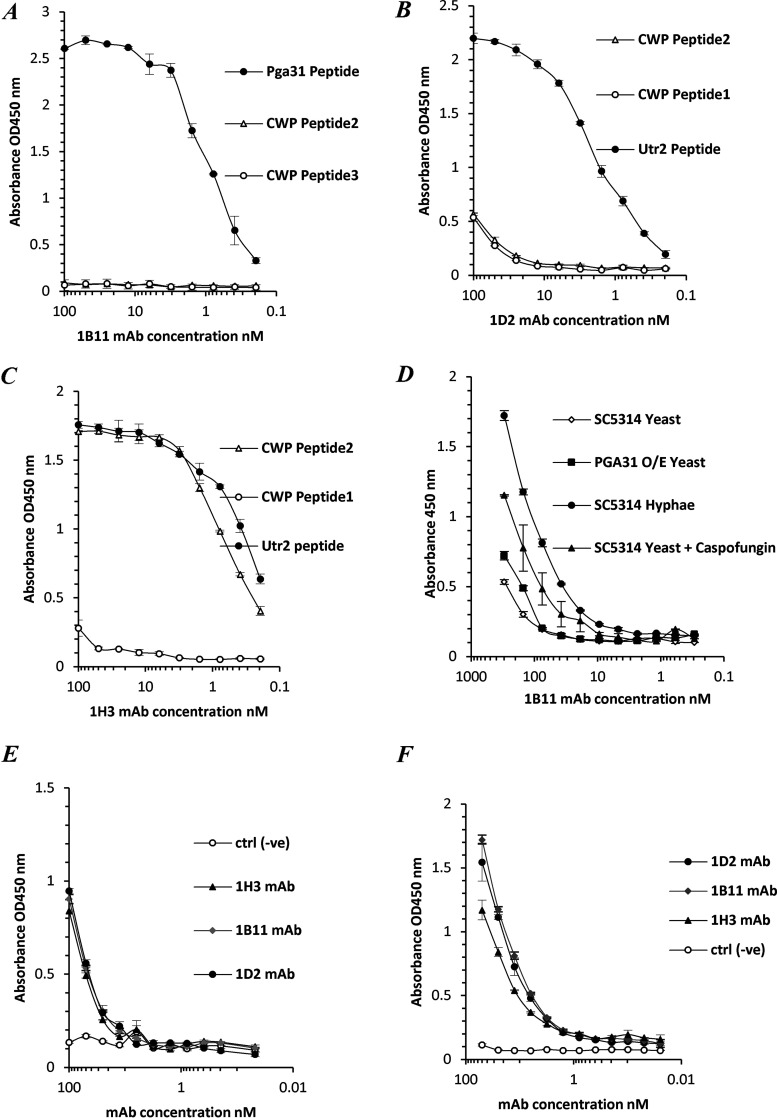
Reformatted Pga31 and Utr2 mAb binding profiles. (A) 1B11 mAb binding to streptavidin-captured biotinylated Pga31 peptide while also assessing cross-reactivity for other C. albicans cell wall proteins (CWP2 and CWP3) using peptide antigens isolated following proteome analysis. (B and C) 1D2 and 1H3 mAb binding to streptavidin-captured biotinylated Utr2 peptide and assessment of cross-reactivity for other C. albicans cell wall protein peptide antigens as described above. (D) 1B11 mAb binding to C. albicans SC5314 yeast cells, a *PGA31*-overexpressing strain (O/E), C. albicans SC5314 hyphae, and C. albicans SC5314 yeast cells treated with 0.032 μg/mL caspofungin. (E) 1B11, 1H3, and 1D2 mAbs binding to C. albicans SC5314 yeast cells. ctrl (−ve), negative control. (F) 1B11, 1H3, and 1D2 mAbs binding to C. albicans SC5314 hyphae. The binding of mAbs to C. albicans peptides and whole cells was detected and quantified using an anti-mouse IgG Fc region-specific HRP-conjugated secondary antibody, and the values plotted represent mean absorbance readings (OD_450_) (*n* = 2 [samples run in duplicate]). Error bars indicate the SEM.

Utr2 scAbs 1D2 and 1H3 and Pga31 scAb 1B11 were selected for IgG reformatting based on their binding interactions with target peptides and C. albicans cells and their protein expression levels. The VH and VL domain genes of the Utr2 scAbs 1D2 and 1H3 and the Pga31 scAb 1B11 were cloned into a dual-plasmid eukaryotic expression system encoding mouse IgG2a and kappa constant domain genes, and the resultant recombinant chimeric mAbs were expressed transiently in HEK293-F cells. The presence of functional, protein A affinity-purified mAbs 1B11 and 1D2 was confirmed by an antigen binding ELISA, with no cross-reactivity to unrelated peptide sequences being observed (Fig. 3A and B). mAb 1H3, which was selected against the Utr2 peptide, appeared to also recognize a peptide sequence selected as a surface-exposed region from the C. albicans cell wall protein Phr2 (Fig. 3C). The reason for this is unclear.

The conversion of scAbs into a bivalent IgG format significantly increased (possibly in part via avidity) the relative binding affinities of all three lead antibodies. Their 50% effective concentration (EC_50_) (antibody concentration required to achieve a 50% reduction in the maximum absorbance) values were calculated by extrapolating values obtained from direct antigen binding plots (Fig. 1A, Fig. 2A, and Fig. 3A to C). The calculated EC_50_ for the 1H3 scAb from the peptide binding assay was 175 nM, whereas the reformatted 1H3 mAb achieved half-maximal binding at 400 pM, an apparent 400-fold improvement in functional affinity. Similarly, the EC_50_ values obtained for the 1D2 scAb and mAb were 80 nM and 2 nM, respectively. For the Pga31 clone 1B11, mAb reformatting resulted in a 600-fold improvement in antigen binding compared to the parental scAb clone, with estimated EC_50_ values of 600 pM and 375 nM, respectively.

In a whole-cell binding ELISA, Pga31 mAb 1B11 was seen to recognize both wild-type C. albicans (SC5314) hyphal and yeast forms (Fig. 3D). Although relatively low binding activity was observed for the yeast form, when the cells were stressed with caspofungin treatment, an enhancement in mAb targeting was seen (Fig. 3D). The 1H3 and 1D2 mAbs were able to bind SC5314 cells immobilized on maxisorbent plates, with similar levels of immunoreactivity being observed in the yeast and hyphal forms (Fig. 3E and F).

### Immunofluorescence staining of C. albicans using Utr2 and Pga31 antibodies.

**FIG 4:**
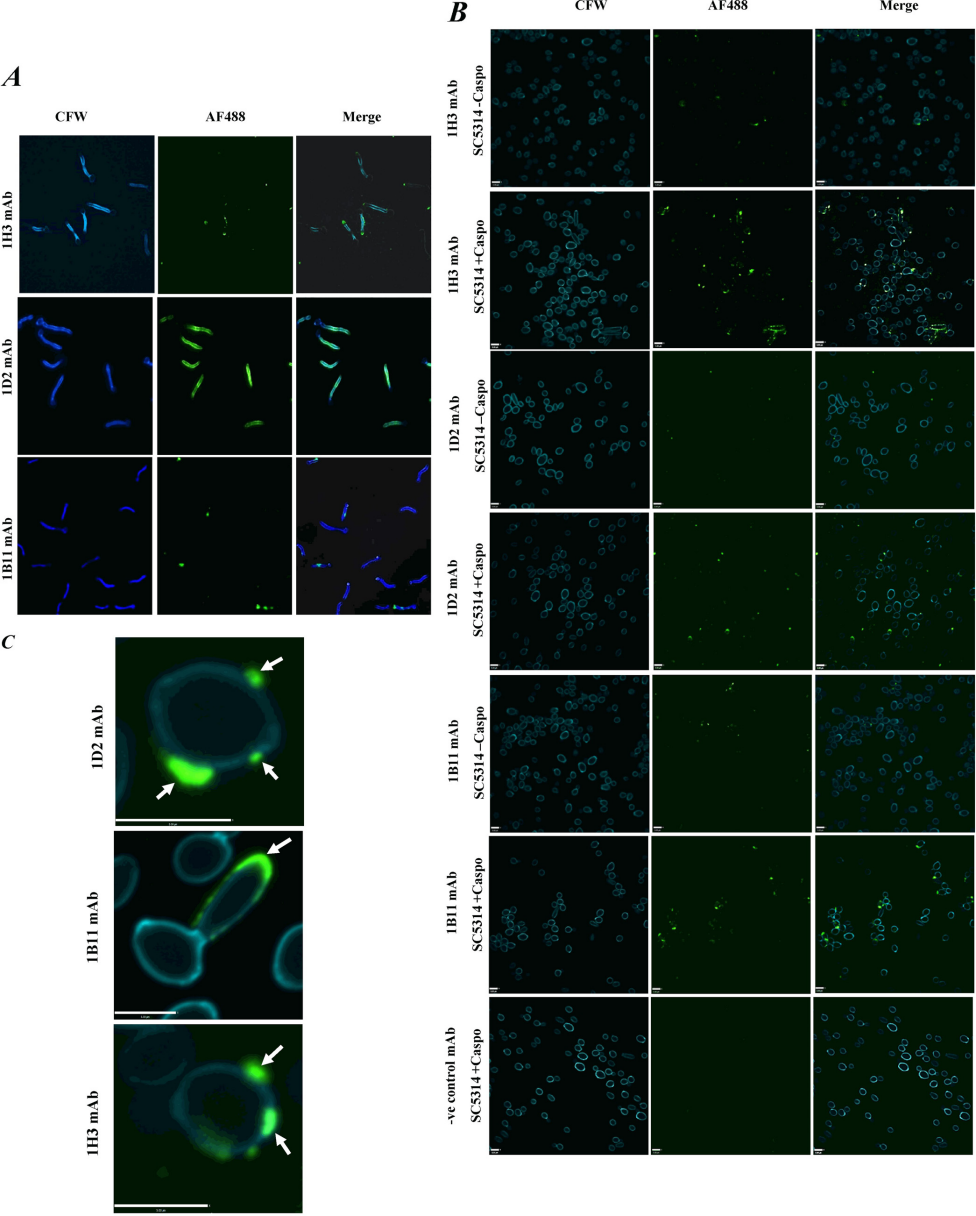
Utr2 and Pga31 antibody binding to C. albicans yeast cells and hyphae. (A) C. albicans SC5314 hypha immunostaining with 1H3, 1D2, or 1B11 antibodies. Calcofluor white (CFW) was used to stain cell wall chitin, and Alexa Fluor 488 (AF488)-conjugated goat anti-mouse IgG2a antibody was used to detect CWP-specific mAb binding. Green fluorescence indicates antibody binding on the cell surface, and distinct binding patterns were observed with the three test mAbs. The anti-Utr2 antibody 1H3 binds at the apical tips of growing hyphae. In contrast, the second Utr2 mAb, 1D2, displayed uniform binding along the hyphae. The anti-Pga31 mAb 1B11 had a more localized binding pattern, with binding to a single major location on the growing hyphae. (B) C. albicans SC5314 yeast cells treated with or without caspofungin (0.032 μg/mL) and immunostained with 1H3, 1D2, or 1B11 antibodies or a negative-control mouse Ig2a antibody. Increased mAb binding was observed in caspofungin-treated cells, mostly as a punctate binding pattern around the poles of buds. (C) The anti-Utr2 mAb 1D2 displays distinct binding, with intense staining localized around zones of polarized growth and away from the mother cell. The second anti-Utr2 mAb, 1H3, and anti-Pga31 mAb 1B11 showed punctate binding on the cell surface (indicated by white arrows).

Immunofluorescence microscopy using cell wall protein-specific antibodies 1D2, 1H3, and 1B11 demonstrated specific and distinct binding patterns on C. albicans cells. Antibodies 1B11 (anti-Pga31) and 1D2 (anti-Utr2) bound to C. albicans SC5314 hyphae but exhibited little or no binding to mother yeast cells (Fig. 4). The enhanced binding in *Candida* sp. hyphae suggests a morphology-dependent binding function, which could signify increased epitope accessibility in this phenotype. 1D2 staining was visible across the entire hyphal surface, whereas 1B11 showed more punctate binding in distinct hyphal regions (Fig. 4A). The cross-reactive 1H3 mAb (anti-Utr2) appeared to bind specifically to the apical tip of growing hyphae (Fig. 4A). When C. albicans SC5314 yeast cells were stained using anti-Utr2 and anti-Pga31 mAbs, a punctate binding pattern was observed on the surface of a limited number of cells (Fig. 4B). In contrast, when yeast cells were pretreated with 0.032 μg/mL caspofungin, strong binding, again punctate in nature, was seen in a large proportion of cells at multiple sites, including regions of possible bud emergence (Fig. 4C). Similar to hyphal staining, the 1D2 mAb produced a distinct binding pattern in budding yeasts, where staining was localized to the emerging daughter cells in areas where a new cell wall was being produced (Fig. 4C). The negative-control mAb did not show any staining of caspofungin-treated or untreated cells (Fig. 4B). In summary, all three antibodies displayed a morphology-specific binding pattern, including increased binding to yeast cells treated with caspofungin, supporting the above-described ELISA data (Fig. 3).

### Macrophage interaction assay.

To evaluate the ability of CWP-specific mAbs to potentially confer protection in an infection model, a macrophage interaction assay was performed using anti-Pga31 1B11 or anti-Utr2 mAb 1H3 as an opsonizing agent for immune cell recruitment and mediation of phagocytosis. Anti-Utr2 mAb 1H3 was chosen due to its cross-reactive nature and peculiar binding pattern by immunofluorescence staining. C. albicans SC5314 yeast cells that were either untreated or precoated with test mAbs or a commercially sourced anti-*Candida* IgG were used to challenge mouse J774.1 macrophage-like cells. The outcomes were visualized by live-cell video microscopy. The engulfment time (the time taken for the macrophages to engulf C. albicans cells) and the length of intracellular hyphae were determined.

Cells preincubated with mAbs and the mouse IgG control were engulfed significantly more rapidly than C. albicans cells without antibody pretreatment (Table 3). The vast majority (95%) of fungal cells were engulfed within 7 min, compared to 10 min for untreated cells (see Fig. S1A to D in the supplemental material). When incubated with anti-Pga31 or anti-Utr2 mAbs, all fungal cells were engulfed by 12 min; however, in the case of untreated C. albicans, this took 15 min (Fig. S1A). These data suggest that anti-CWP mAbs influence macrophage behavior by targeting fungal cells for opsonophagocytosis.

**TABLE 3:** Time taken for J774.1 mouse macrophages to engulf anti-*Candida* mAb-treated or untreated C. albicans SC5314 cells^*a*^

Treatment group	Mean avg engulfment time (min) ± SD
SC5314 (wild type)	5.64 ± 2.59
SC5314 + anti-Pga31 mAb	4.18 ± 1.80
SC5314 + anti-Utr2 mAb	4.28 ± 1.96
SC5314 + IgG positive-control mAb	4.29 ± 2.43

a At least 25 macrophages were selected at random per video to determine the engulfment time. Data represent the average time taken ± SD (minutes).

**FIG 5:**
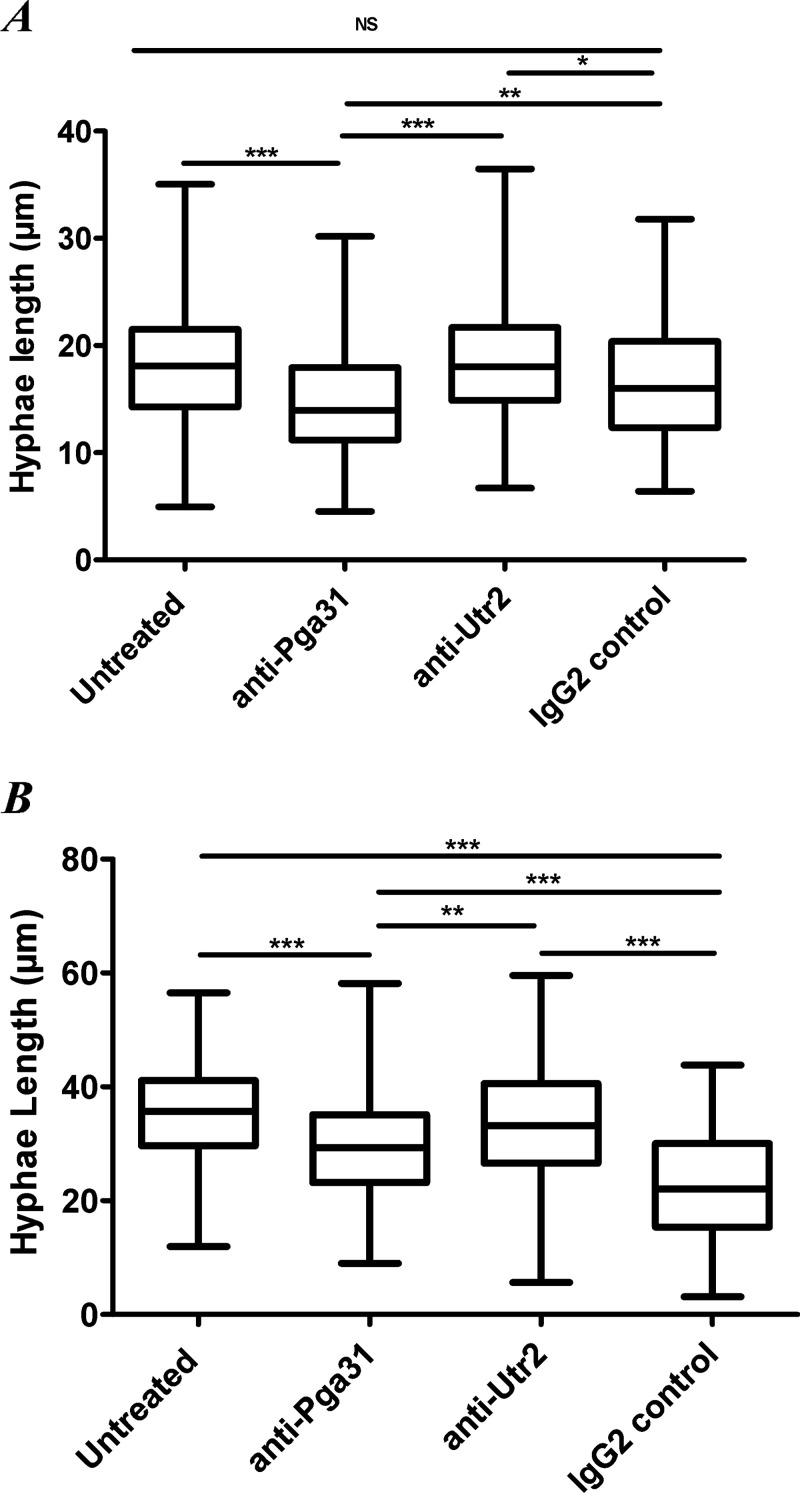
Lengths of intracellular hyphae at 60 and 90 min. Intracellular hyphal lengths were measured (micrometers) following C. albicans uptake by J774.1 mouse macrophages at 60 min (A) and 90 min (B). Twenty-five macrophages were selected at random per video to measure the length of intracellular hyphae at two time points. Statistical significance was determined by a Kruskal-Wallis test with Dunn’s multiple-comparison test. *, *P* < 0.05; **, *P* < 0.01; ***, *P* < 0.005; NS, not significant.

The length of intracellular hyphae at multiple incubation times was also analyzed from microscopy videos (Fig. 5). Measurements were taken from the neck of the hypha to the apical tip 60 and 90 min following coincubation with macrophages. Our data show that intracellular hyphae at 60 min were significantly shorter for C. albicans cells preincubated with anti-Pga31 mAb than for all other treatment groups (*P* ≤ 0.0001) (Fig. 5A). C. albicans cells pretreated with the positive-control IgG were also shorter than untreated cells, but the difference was significant only at 90 min (Fig. 5A and B).

### Testing the therapeutic efficacy of anti-Utr2 and anti-Pga31 mAbs in a C. albicans mouse infection model.

**FIG 6:**
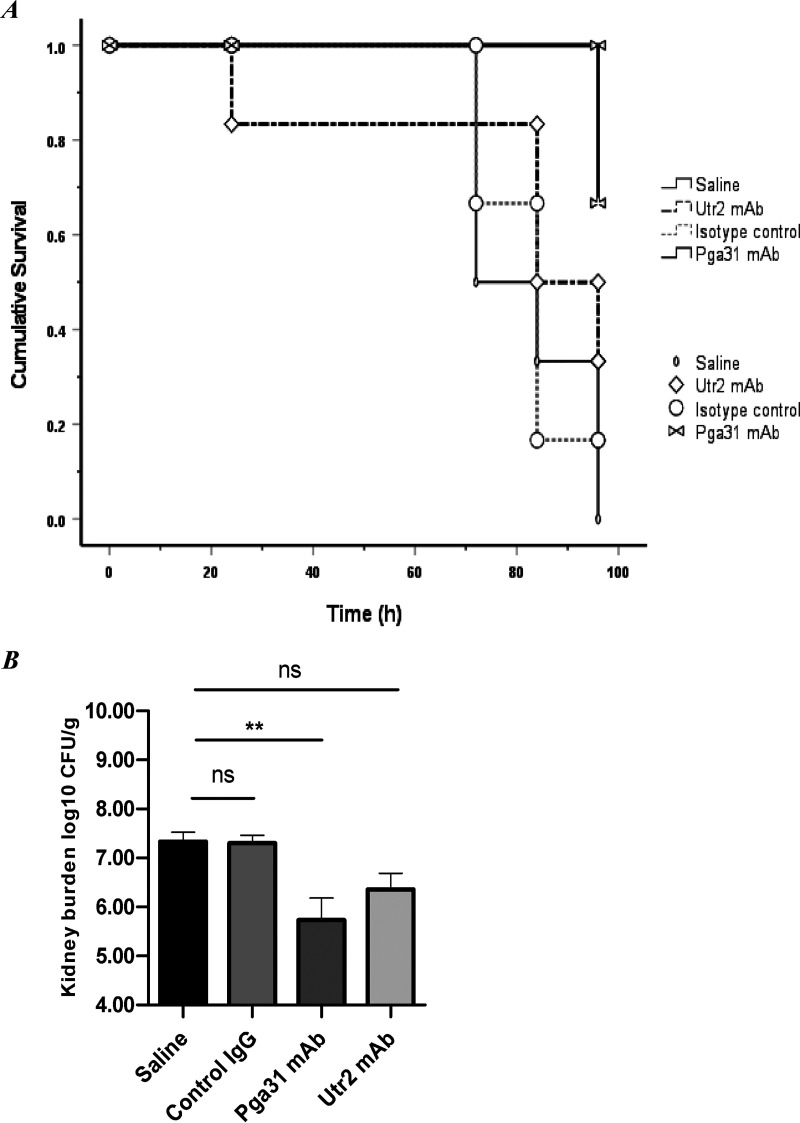
(A) Kaplan-Meier survival curve representing the treatment effects of test mAbs and control groups over 96 h in study 1. Pga31 mAb 1B11 (15 mg/kg), Utr2 mAb 1D2 (15 mg/kg), isotype control IgG (15 mg/kg), or saline was administered i.p. to mice 3 h before and 24 h after infection with C. albicans SC5314 (i.v. administration of 2.4 × 10^4^ to 2.6 × 10^4^ CFU/g body weight in 100 μL). Mice (*n* = 6/group) surviving the study period were treated as censored data for analysis, and the statistical significance of survival between groups was determined using the log rank test. (B) Mean fungal burdens in kidneys on day 4 postinfection in study 1. Isotype control IgG (15 mg/kg i.p.), Pga31 mAb 1B11 (15 mg/kg i.p.), and Utr2 mAb 1D2 (15 mg/kg i.p.) were administered 3 h before and 24 h after infection with C. albicans SC5314 (i.v. administration of 2.4 × 10^4^ to 2.6 × 10^4^ CFU/g body weight in 100 μL). Error bars denote standard deviations. A significant difference was observed for kidney burdens between the saline treatment group and the 1B11 mAb therapy group (*n *= 6 mice/group) (*P* = 0.004 by a Kruskal-Wallis test with Dunn’s multiple-comparison test). **, *P*, 0.01; ns, not significant.

A series of *in vivo* mouse infection studies was conducted to evaluate the protective effect of Pga31 and Utr2 mAbs in a disseminated candidiasis model, with efficacy being measured by determining the organ fungal burden and mouse survival. In prophylactic study 1, mice were pretreated with 15 mg/kg of body weight of the test mAbs (including an isotype control), 3 h prior to the intravenous (i.v.) administration of C. albicans SC5314, followed by a second dose of mAb at 24 h postinfection. All treatments were administered intraperitoneally (i.p.) in 150 μL saline. In the anti-Pga31 mAb 1B11-treated group, 67% of mice survived to 4 days postinfection, whereas Utr2 mAb 1D2 conferred 33% protection (Fig. 6A). Treatment with the isotype control did not show any survival benefit compared to the saline group at 4 days. Comparing across all groups, there were significant differences in survival (*P *= 0.045 by Kaplan-Meier log rank statistics).

Comparing the percentage weight changes (days 0 to 2) (Table S1) and kidney fungal burdens (Fig. 6B), the differences were again statistically significant between some groups (*P* < 0.001 by a Kruskal-Wallis test with Dunn’s multiple-comparison test). In particular, comparing the fungal kidney burden of the saline-only group to that of the Pga31 mAb-treated group (*P *= 0.004) and weight changes (*P *= 0.011), the Utr2 treated group showed a significant difference for weight change only (*P *= 0.023). There was no difference in kidney fungal burdens between the isotype control IgG-treated mice and the saline-treated mice (*P *> 0.999).

**FIG 7:**
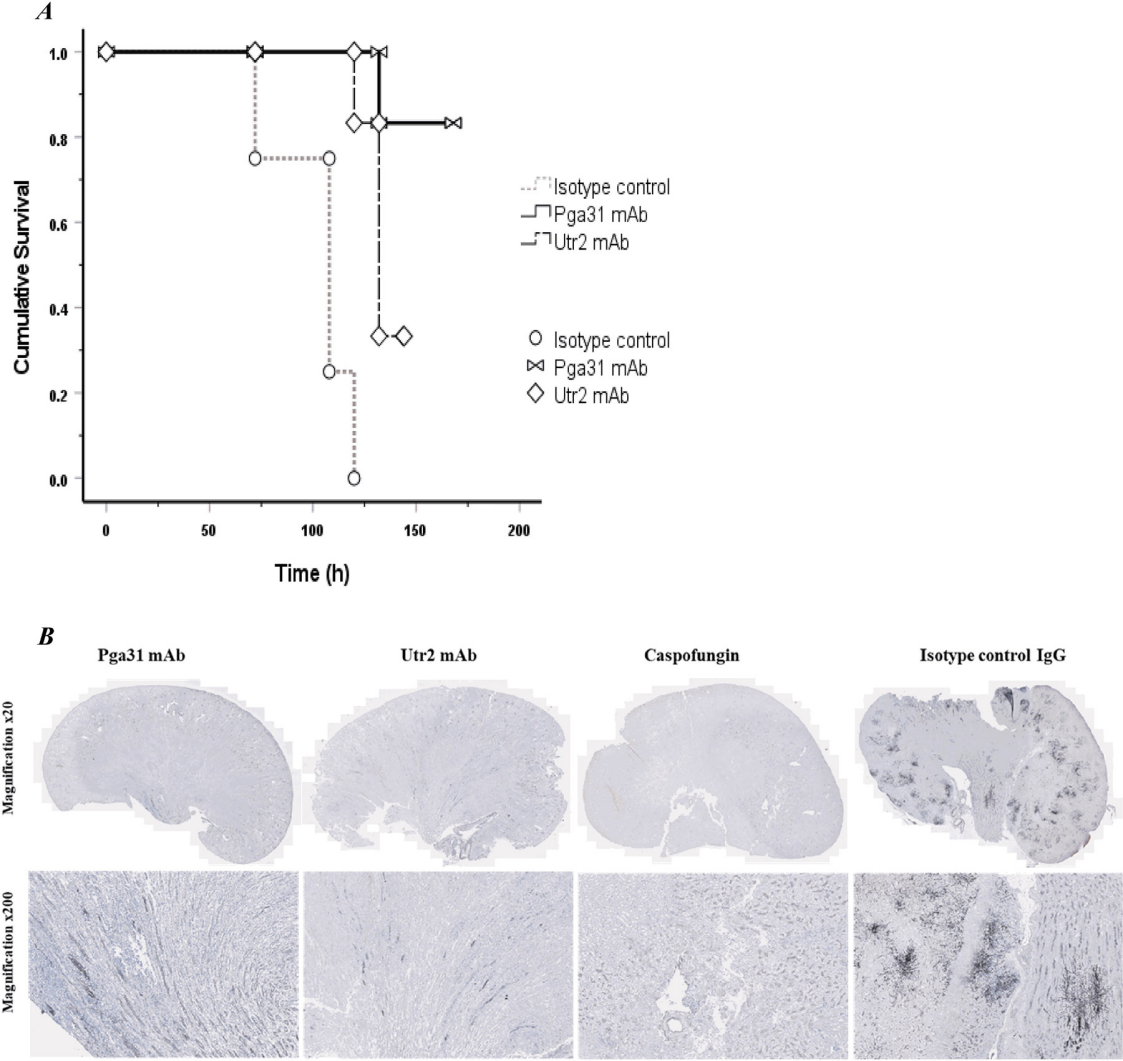
(A) Kaplan-Meier survival curve representing the treatment effect of test mAbs and control groups 6 days post-infection (Study 2). Pga31 mAb 1B11 (12.5 mg/kg), Utr2 mAb 1D2 (12.5 mg/kg) or isotype control IgG (12.5 mg/kg) were administered IP in mice 3 h pre- and 24 h and 48 h post-infection with *C. albicans* SC5314 (IV administration; 2.1-2.3 × 104 CFU/g body weight in 100 μl). The difference between the three groups (*n* = 6 mice/group) is statistically highly significant (*P* < 0.001, Kaplan-Meier log-rank test). (B) Representative histopathological sections of kidneys of mice treated with Pga31 mAb, Utr2 mAb, isotype control IgG or caspofungin (1 mg/kg). Tissue sections were stained using Gomori’s methenamine silver (GMS) method. Numerous fungal lesions are seen in the isotype control treated kidney (black staining) compared to kidneys from the mAb treatment groups and caspofungin group.

A second study was conducted to test the effectiveness of multiple dosing of antibodies. A single 12.5-mg/kg dose was administered prophylactically, followed by two treatment doses at 24 and 72 h postinfection. All treatments were administered i.p. in 150 μL saline, and the survival rates of mice treated with Pga31 and Utr2 mAbs were compared with those of mice treated with an isotype control. Mice were monitored and weighed every day and culled on day 6 postinfection, and fungal burdens in several organs, including the kidneys, brain, and spleen, were determined as described above. While a mouse survival rate of 83% was achieved with the Pga31 mAb following a second dose at 72 h postinfection (versus 66.7% for study 1), no further improvement was observed for the Utr2 mAb (33% survival in studies 1 and 2) (Fig. 7A). Isotype control IgG showed no therapeutic effect, and the differences between various groups are statistically significant (*P* < 0.001 by a log rank test). A 1-log drop in the kidney fungal burden, representing killing of fungi or inhibition of cell division, for the Pga31 mAb-treated group was achieved compared to the isotype control group (Pga31 mAb = 5.5. log_10_ CFU/g; isotype control = 6.8 log_10_ CFU/g); however, there was little or no difference in mean fungal counts for the Utr2 mAb-treated group (Utr2 mAb = 6.7 log_10_ CFU/g; isotype control = 6.8 log_10_ CFU/g) (Table 4). Similarly, the fungal burden in associated organs, including the brain and spleen, was also reduced in the test antibody groups, with the Pga31 mAb showing an improved therapeutic effect compared to the Utr2 mAb.

**TABLE 4:** Mean fungal burdens in mouse organs at 6 days postinfection (study 2)^*a*^

Treatment	Mean log_10_ CFU/g of organs ± SD from disseminated candidiasis model (*n* = 6 mice/group)		
	Kidney	Spleen	Brain
Isotype control	6.8 ± 0.7	4.0 ± 0.5	4.8 ± 0.4
Pga31 mAb	5.5 ± 0.5	3.3 ± 0.1	3.4 ± 0.7
Utr2 mAb	6.7 ± 0.7	3.6 ± 0.4	4.6 ± 1.0

a Control IgG (12.5 mg/kg), Pga31 mAb 1B11 (12.5 mg/kg), and Utr2 mAb 1D2 (12.5 mg/kg) were administered i.p. 3 h before infection and 24 h and 72 h after infection with C. albicans SC5314 (i.v. administration of 2.1 × 10^4^ to 2.3 × 10^4^ CFU/g body weight in 100 μL) (*n* = 6 mice/group). Statistical significance was achieved for fungal counts in the kidneys only (*P* = 0.02 by Kruskal-Wallis and Dunn’s multiple-comparison tests).

**FIG 8:**
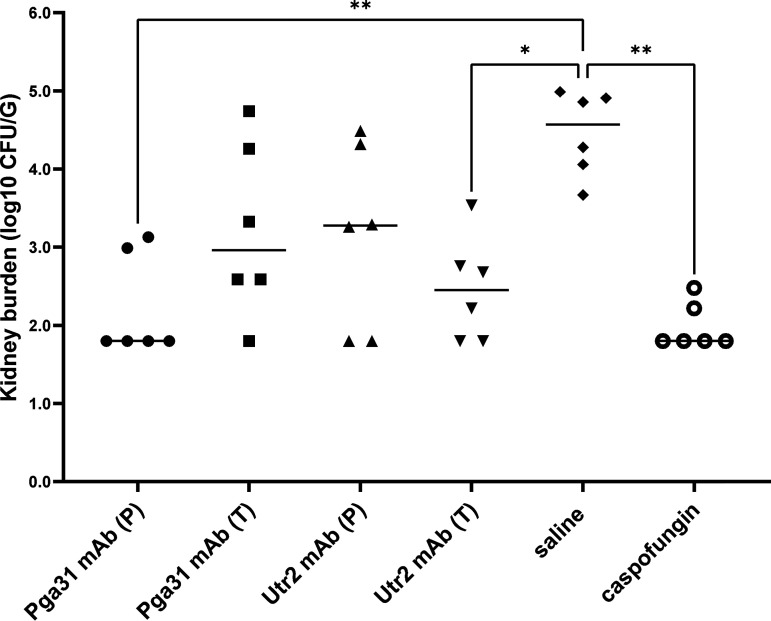
Kidney burdens at day 7 postinfection in the prophylactic-versus-treatment study. Test mAbs (12.5 mg/kg per mouse) were administered 3 h before infection and/or 24 h and 72 h after infection with C. albicans SC5314 (i.v. administration of 2.0 × 10^4^ to 2.5 × 10^4^ CFU/g body weight in 100 μL) (*n *= 6 mice/group). Pga31 mAb (P), prophylactic arm, with antibody given pre- and postinfection; Pga31 mAb (T), treatment arm, with antibody given postinfection only; Utr2 mAb (P), prophylactic arm; Utr2 mAb (T), treatment arm; saline, saline-only control; caspofungin, caspofungin at 1 mg/kg body weight at 24 h and 72 h postinfection. Each symbol represents an individual mouse, and bars represent the mean kidney burdens in each group. The detection limit for kidney burden determination was 2.3 log_10_ CFU/g, and therefore, any samples with zero counts were assigned a value of 1/2 log below the detection limit (i.e., 1.8 log_10_ CFU/g). Kidney burdens for the different groups were compared by Kruskal-Wallis and Dunn’s multiple-comparison tests. *, *P* < 0.05; **, *P* < 0.01; ***, *P* < 0.005.

Finally, to compare the protective effect of test antibodies as a prophylactic agent versus treatment, a follow-up study (study 3) was conducted. In the prophylactic arm, a single dose of each test antibody was administered 3 h before infection, followed by two doses at 24 h and 72 h postinfection. For the treatment-only arm, mAbs were given at 24 h and 72 h postinfection, and the fungal burdens in the kidneys of various groups were compared to those in groups of mice receiving saline or caspofungin (1 mg/kg) (Fig. 8). The Pga31 mAb prophylactic arm significantly reduced the fungal burden in the kidneys of animals, at 7 days postinfection, which was similar to the levels achieved with caspofungin treatment (Pga31 mAb = 2.22 log_10_ CFU/g; caspofungin = 1.98 log_10_ CFU/g; saline = 4.46 log_10_ CFU/g [*P *= 0.002 by a Kruskal-Wallis test]). Mice receiving only two doses of Pga31 mAb postinfection also had reduced fungal burdens in their kidneys (3.22 log_10_ CFU/g). Interestingly, for the Utr2 mAb groups, the treatment arm where the test antibody was administered at 24 h and 72 h postinfection had a reduced burden in the kidneys as opposed to the prophylactic arm (Utr2 mAb treatment arm = 2.47 log_10_ CFU/g; prophylactic arm = 3.16 log_10_ CFU/g).

## DISCUSSION

With the advent of mAb technology, several groups have reported the development of protective antibodies as a central part of a patient’s recovery from infection (30). These mAbs typically recognize antigens that are unique to the fungus and include fungal cell wall polysaccharides and a small number of cell wall proteins (Als3, Sap2, Hsp90, and Hry1) involved in cell growth, virulence, and pathogenesis, as reviewed previously (15, 31–33). Utr2 and Pga31 are CWPs covalently linked to the fungal cell wall, with their levels of expression being affected by the carbon source (34) and infection-associated stress conditions, including external stimuli such as challenge with antifungal agents (35). We have recently reported the increased expression of Utr2 and Pga31 proteins, at proteomic levels, in C. albicans grown in the presence of caspofungin (22). In most cases, the C-terminal ends of GPI-anchored proteins are buried inside the β-glucan skeletal layer, with only stretches of amino acids at the N-terminal functional domain being surface exposed (36). Surface epitopes of Utr2 and Pga31 were deduced from tryptic peptides generated from our cell wall proteome studies and recombinant mAbs isolated that recognized CWPs in their native conformation (Fig. 3D to F). Pga31 antibodies showed increased binding to C. albicans whole-cell lysates grown in the presence of caspofungin (Fig. 1C and D), reaffirming observations that antifungal agents alter CWP expression (22) and that Pga31 is expressed as a remodeling mechanism for maintaining wall integrity under cellular stress. A role for Utr2 in establishing a compensatory mechanism for the cross-linking of chitin and β1-3 glucan in echinocandin-treated cells was reported previously (18). The abundance of the Utr2 protein in the wall of caspofungin-treated cells is confirmed here, with increased antibody binding to cell lysate preparations (Fig. 2D to F). The target specificity of these antibodies was confirmed by the lack of binding to mutant strains (*utr*2Δ and *utr*2Δ/*crh11*Δ/*crh12*Δ), even in the presence of caspofungin (Fig. 2G).

We observed C. albicans cell morphology-dependent immunoreactivity of CWP antibodies, with enhanced binding to the hyphal form compared to yeast cells (Fig. 4A and B). Utr2 mAb 1D2 bound uniformly along the growing hyphae, indicating broad surface exposure of this antibody’s preferred epitope. In contrast, the second Utr2 mAb, 1H3, displayed a different binding pattern, being localized mostly to the apical tip of growing hyphae, suggesting the recognition of a second and distinct epitope at the tip of the germ tube during hyphal elongation. The significance of the Crh family of proteins, including Utr2, in cell wall biogenesis and their temporal and spatial organizations in various morphologies have been elegantly reported previously (18). The present study supports the idea that Utr2 accumulation initially is localized to new budding sites in yeast during the early growth phase, followed by relocalization toward the base of the bud neck, overlapping the chitin ring later in the cell cycle. 1H3 and 1D2 mAb binding to cells pretreated with caspofungin also saw the greatest signal intensity at the new bud surface, further supporting this finding.

Pga31 mAb binding resulted in a weaker but punctate signal in distinct hyphal regions of C. albicans cells. This binding pattern is in agreement with previous reports and our finding that Pga31 is expressed at low or undetectable levels in C. albicans under normal laboratory growth conditions, e.g., in yeast extract-peptone-dextrose (YPD) culture medium. Pga31 is suggested to be part of the cell salvage pathway (16) and regulates chitin assembly when cells are treated with cell wall-perturbing agents, including calcofluor white and caspofungin (12). In our study, a marked increase in Pga31 binding was also observed when cells were treated with caspofungin (Fig. 4B).

The ability of J774.1 macrophages to engulf C. albicans cells treated with CWP-specific mAbs or an isotype control anti-*Candida* mAb was significantly greater than that of non-antibody-treated cells (Table 3). A complex interplay between macrophage and C. albicans has been previously reported, with the pathogen sometimes counteracting the macrophage’s defense strategies to eventually break free and kill the macrophage as it escapes (37). In our study, while antibody-mediated engulfment did not result in the complete killing of C. albicans, significant inhibition of hyphal filamentation was observed, which is tempting to speculate is due in part to an immunomodulatory activity of mAbs involved in pathogen clearance. However, the level of macrophage activity, while real, was modest, yet the performance of our mAbs *in vivo* was highly potent. Therefore, it makes sense to speculate that in addition to macrophage clearance, antibody-mediated recruitment of neutrophils, natural killer cell-directed cytotoxicity, and complement fixation may also play prominent roles in fungal killing (32). It has been known for some years now that neutrophils can prevent C. albicans yeast-to-hypha conversion and can act as potent effector cells for pathogen clearance by FcγR activation, facilitation of phagolysosomal killing by the generation of reactive oxygen species (ROS), and other candidacidal granular proteases (38).

mAb-mediated pathogen clearance was most convincingly demonstrated *in vivo*, in a disseminated candidiasis mouse model, where protection from a life-threatening infection was evident in animals receiving CWP-specific mAbs compared to an isotype control mAb or the vehicle alone. The Pga31 mAb, in particular, when administered as a single dose preinfection followed by two doses at 24 h and 72 h postinfection, conferred improved survival rates (compared to a single dose pre- and postinfection) of 83% versus 66%, respectively. The benefit of double dosing was also reflected in the kidney fungal burdens, with a very respectable 3-log reduction (99.9%) in the number of fungal cells seen in the kidneys of mice receiving two doses of mAb after infection.

Other published *in vivo* efficacy models typically describe test mAbs that were either preincubated with C. albicans cells or administered as a prophylactic before pathogen challenge, with survival benefits and any reduction of fungal burden in associated organs reported (26, 39). With these experimental design parameters, mAbs are already present in the systemic circulation and able to bind to yeast cells, mediating opsonophagocytosis and clearance with enhanced protection. Survival rates of between 40% and 50% in a mouse model of systemic candidiasis have been claimed for the β-(1→3)-d-glucan mAbs (39), and in a separate study, a <1-log reduction in kidney fungal burdens was reported for an anti-C. albicans mAb isolated from patient B cells (26). Using a peptide biologic therapy, rather than a much larger antibody, a small protective effect in animal studies with only a 1-log drop in the fungal burden was seen in “topical” vaginal and oropharyngeal candidiasis models (40). This less potent systemic efficacy may be the result of the peptides’ “sticky” mode of action, low bioavailability, or significantly reduced half-life compared to mAbs (41, 42). Interestingly, in our study 3 design, CWP-specific antibodies also reduced the fungal burdens in the kidneys of mice receiving treatment at 24 h postinfection, providing an early indication of their ability to bind *in vivo* to cells with both yeast and hyphal morphologies, possibly inhibiting cell replication and/or enhancing phagocytosis and clearance. In the case of the anti-Utr2 mAb, double-dose treatment did not translate into increased survival compared to the single dose (33% in both studies 1 and 2). But in study 3, specifically investigating kidney fungal burdens, two doses of Utr2 mAb in the treatment-only group were more protective than mAb given to mice as a prophylactic followed by two doses postinfection. This observation warrants further experimental validation, especially in the context of using Utr2 mAb as a therapeutic intervention after an infection has been established.

With the serum half-lives of therapeutic human IgGs often being reported to be in the region of 21 to 28 days (a few days in mice) (43), immunotherapy for invasive fungal infections can reduce the dosing frequency while addressing the serious drug resistance issues associated with long-term treatment regimens adopted for chronic infections. This is particularly pertinent for non-*albicans Candida* species, including Candida krusei, C. glabrata, and C. auris, which are either intrinsically tolerant or fully resistant to one or more classes of existing antifungals, increasing the prevalence of nontreatable nosocomial infections (44). Off-target toxicity and drug-drug interactions are other important treatment considerations associated with existing antifungal therapies. Each drug class has its clinical challenges: nephrotoxicity of polyenes (45), amphotericin B interactions with hypokalemic drugs resulting in cardiac and skeletal muscle toxicity, and azole-mediated inhibition of metabolizing liver enzymes (e.g., cytochrome P450) leading to decreased catabolism of coadministered drugs and dose-related toxicities (46). It is widely recognized that targeted biological agents, such as monoclonal antibodies, can overcome many of the toxicity and drug resistance hurdles created by using small-molecule drugs, with a few mAbs now approved by the FDA as prophylactic treatments against bacterial and viral infections (47). One could easily envisage scenarios where patients undergoing chemotherapy or organ transplant might receive long-acting, antifungal mAbs as prophylaxis against life-threatening invasive fungal infections prevalent in these immunocompromised groups. These mAbs could be used as part of a cotherapy regimen to augment or prolong the activity of existing, but limited-in-number, systemic antifungals and address the issue of drug resistance, especially with fungistatic azole compounds. By simultaneously targeting the pathogen with an antifungal agent and a mAb adjuvant, the same level of efficacy can be achieved with lower drug doses, thereby significantly improving the narrow therapeutic window associated with commonly used antifungals.

In the present study, we successfully identified mAbs as lead molecules in a new antifungal drug class as the first step in providing alternatives to our current and very limited antifungal drug portfolio. A panel of recombinant human antibodies capable of recognizing surface-exposed epitopes of key fungal cell wall proteins (in a range of fungal pathogens) has been isolated and characterized. These antibodies have been selected to recognize target proteins in their native conformation and confer excellent levels of protection (over 80% with Pga31 antibody 1B11) in a mouse model of disseminated candidiasis, in part through the recruitment of phagocytic macrophages via antibody-mediated opsonization. These novel antifungal mAbs are now entering later-stage preclinical evaluation to investigate additional aspects of their mode of action, levels of *in vivo* tolerability, and pharmacological activities, including pharmacokinetic/pharmacodynamic (PK/PD) profiles, in relevant animal models.

## MATERIALS AND METHODS

### Fungal strains, media, and growth conditions.

All fungal strains used in this study are shown in Table 5 and were cultured from glycerol stocks (−70°C) and maintained on YPD agar plates containing 2% (wt/vol) glucose, 2% (wt/vol) mycological peptone, 1% (wt/vol) yeast extract, and 2% (wt/vol) agar (all from Oxoid, Cambridge, UK). For C. albicans strains, unless stated otherwise, a single colony was grown in YPD medium (as described above but without the agar) and grown overnight at 30°C with shaking at 200 rpm. To induce hypha formation, cells were grown in RPMI 1640 modified medium (Sigma-Aldrich) containing 10% heat-inactivated fetal calf serum (FCS) and incubated at 37°C for 2 to 4 h. For mouse studies, C. albicans SC5314 was grown in NGY medium containing 0.1% Neopeptone (BD, Wokingham, UK), 0.4% glucose, and 0.1% yeast extract (BD) at 30°C with constant rotation at 200 rpm.

**TABLE 5:** C. albicans strains used in this study

Strain	Description	Genotype	Reference
SC5314	Clinical isolate	Wild type	50
GPY03	*utr2*Δ	*ura3*Δ::λ*imm434*/*ura3*Δ::λ*imm434 utr2*Δ::*hisG*/*utr2*Δ::*hisG-URA3-hisG*	18
GPY102	*crh11*Δ/*crh12*Δ/*utr2*Δ	*ura3*Δ::λ*imm434*/*ura3*Δ::λ*imm434 utr2*Δ::*hisG*/*utr2*Δ::*hisG*; *crh12*Δ::*hisG*/*crh12*Δ::*hisG*; *crh11*Δ::*hisG crh11*Δ::*hisG-URA3-hisG*	18
CAMY 204	*UTR* overexpression strain	*ura3*Δ::λ*imm434*/*ura3*Δ::λ*imm434 his1*Δ::*hisG*/*HIS1 arg4*Δ::*hisG*/*ARG4 ADH1*/*ADH1*/*adh1*::*P_ADH1_-cartTA*::*SAT1 RPS1*/*rps1*::*CIp10-GTW-*P_TET_*-UTR2*	This study
*pga31*Δ	*pga31*Δ	*ura3*Δ::λ*imm434*/*ura3*Δ::λ*imm434 arg4*::*hisG*/*arg4*::*hisG his1*::*hisG*/*his1*::*hisG pga31*::*URA3*/*pga31*::*ARG4*	12
CAM_K46	*PGA31* overexpression strain	*ura3*Δ::λ*imm434*/*ura3*Δ::λ*imm434 his1*Δ::*hisG*/*HIS1 arg4*Δ::*hisG*/*ARG4 ADH1*/*adh1*::*P_ADH1_-cartTA*::*SAT1 RPS1*/*rps1*::*CIp10-GTW-*P_TET_*-PGA31*	This study

### Phage display-based isolation of CWP scFv binders from a human antibody library.

Solution-phase biopanning and monoclonal phage binding enzyme-linked immunosorbent assays (ELISAs) were performed according to previously described methods (48). Briefly, a human antibody library was subjected to repeated rounds of selection using biotinylated peptide antigens corresponding to surface-exposed regions of the cell wall proteins Pga31 (amino acid sequence QPLNVGNTVLQLGGSGDGTKVDIAEDGTLS) and Utr2 (amino acid sequence WPGGDSSNAKGTIEWAGGLINWDSEDIK). In the first round, streptavidin magnetic beads (Dynabeads M280; Invitrogen) were coated with 500 nM biotinylated Utr2 or Pga31 peptide, and phage particles displaying antibody fragments on their surface were incubated for target binding. Phage particles bound to antigen-biotin complexes were eluted with triethylamine (TEA) and amplified by infecting Escherichia coli TG1 cells. For the second and third rounds of panning, the coating concentrations of biotinylated peptide antigen were reduced to 100 nM and 10 nM, respectively, and rescued phage from previous rounds of panning was allowed to bind to the antigen for selection. For screening antigen-specific phage binders, 96-well plates (Nunc MaxiSorp) were precoated with streptavidin to capture biotinylated Pga31 or Utr2 peptides, and the monoclonal phage supernatant was added as described previously (49). A peptide antigen binding ELISA was performed, and individual phage monoclonal antibodies specifically binding to the CWP peptide antigen (and not recognizing a nonrelated biotinylated peptide) were selected for antibody gene sequencing.

### Expression of soluble Pga31 and Utr2 antibody fragments in a bacterial system.

Positive phage clones with unique VH and VL genes were converted into soluble single-chain antibodies (scAbs) by cloning their respective scFv genes (VH-linker-VL) into the bacterial expression vector pIMS147 and transforming E. coli TG1 cells for periplasmic expression as described previously (29). scAbs expressed in the bacterial periplasm were released by adding an osmotic shock solution consisting of Tris-HCl-sucrose and EDTA followed by MgSO_4_, and the mixture was incubated on ice with gentle shaking for 15 min each. Recombinant scAbs were purified using immobilized-metal affinity chromatography (IMAC) columns via the binding of hexahistidine-tagged protein to activated Ni-Sepharose beads and elution using imidazole. Purified scAbs were dialyzed against phosphate-buffered saline (PBS) and quantified either by SDS-PAGE, where the intensities of protein bands were compared (ImageJ), or by calculating final scAb concentrations by measuring the absorbance values at 280 nm using an Ultraspec 6300 pro UV-visible spectrophotometer (Amersham Biosciences).

### Reformatting CWP scAbs into human-mouse chimeric mAbs.

Utr2 and Pga31 scAbs were reformatted into human-mouse (IgG2a) chimeric mAbs by inserting the antibody VH and Vκ genes into a dual-plasmid eukaryotic vector system carrying constant heavy and light chain genes of the mouse IgG2a isotype. VH and Vκ genes of shortlisted scAbs were custom synthesized by introducing the restriction sites BssHII and BstEII (for the VH gene) and BssHII and XhoI (for the Vκ gene) at their 5′ and 3′ ends, respectively (GeneArt custom gene synthesis service by Thermo Fisher), and cloned into the respective eukaryotic expression vectors pEEDM2a (encoding mouse IgG2a constant regions) and pEEDMκ (for the mouse κ constant domain) using standard restriction enzyme digestion and ligation steps. Ligated DNA was purified using ethanol precipitation and used to transform electrocompetent E. coli TG1 cells for plasmid propagation.

### Production of Pga31 and Utr2 mAbs in a mammalian expression system.

For laboratory-scale expression of mAbs, plasmids bearing chimeric antibody heavy and light chain genes were prepared (EndoFree plasmid mega prep kit; Qiagen) and transfected into human embryonic kidney cells (HEK293-F) (Life Technologies) using polyethylenimine (PEI). Transfections were carried out using 1 mg of total DNA (500 μg each of VH and Vκ plasmid DNAs) and 1 L of the cultured HEK293-F cell suspension maintained in sterile Freestyle 293 expression medium (Invitrogen) without antibiotics at 37°C with 8% CO_2_, with shaking at 125 rpm. The transfected cells were grown for 8 days and purified using ProSep A beads (Millipore) and Econo-Pac chromatography columns (Bio-Rad). Recombinant mAbs were eluted in 100 mM glycine (pH 3.0) before neutralization with 1 M Tris-HCl (pH 8.0). Purified mAbs were quantified by SDS-PAGE and *A*_280_ measurements.

### CWP peptide, C. albicans cell lysate, and whole-cell ELISA.

For ELISAs using Pga31 or Utr2 peptides, 96-well Nunc MaxiSorp plates were precoated with 100 nM streptavidin and blocked with 2% Marvel in PBS before adding 500 nM biotinylated peptides. scAb or mAb samples were incubated with the antigen in doubling dilutions, and binding was detected using anti-human C kappa horseradish peroxidase (HRP)-conjugated secondary antibody (Sigma) (for scAbs) or anti-mouse IgG(H+L) HRP secondary antibody (Thermo Scientific) (for human-mouse chimeric mAbs).

For ELISAs of whole yeast cells or total cell lysates using the wt, *pga31*Δ, *crh11*Δ/*crh12*Δ/*utr2*Δ, and *PGA31* overexpression strains of C. albicans, cultures grown overnight were inoculated into fresh YPD medium at a starting optical density at 600 nm (OD_600_) per milliliter of 0.1 to 0.2 and grown at 30°C until an OD_600_ per milliliter of 0.5 to 0.6 was reached, and caspofungin (0.032 μg/mL) was then added for 90 min.

For the preparation of total cell lysates, caspofungin-treated or nontreated cells were harvested, centrifuged for 5 min at 4,000 rpm, and washed with sterile distilled water (dH_2_O) and 10 mM Tris-HCl (pH 7.5) before being resuspended again in fresh Tris-HCl. Glass beads (0.5-mm diameter) were added (0.5 g to each 100-mg pellet) along with a protease inhibitor solution (complete mini EDTA-free protease inhibitor cocktail; Roche), dH_2_O, and 1 mM phenylmethylsulfonyl fluoride (PMSF) in ethanol. Samples were subjected to 15 rounds of bead beating for 35 s at speed 6.5 using a Fast Prep-24 instrument (MP Biomedicals, UK), with tubes being placed on ice for at least 5 min between rounds of bead beating. After centrifugation at 3,000 rpm for 1 min to pellet the beads, the broken cell suspension was transferred to sterile cold tubes. The cell lysate preparation was used to coat ELISA plates as described above.

For ELISAs using *Candida* hyphae, cells were grown in RPMI 1640 medium (for 2 to 4 h) and added to the wells for incubation at 37°C for 1 h. For whole-cell binding ELISAs, *Candida*-coated wells were washed and blocked with 2% bovine serum albumin (BSA; Sigma), followed by the addition of doubly diluted scAb or mAb samples. Binding was detected using anti-human C kappa HRP or anti-mouse IgG(H+L) HRP, and the resulting immunoreaction was measured as described above.

### Immunofluorescence imaging of antibodies binding to fungal cells.

Fungal cultures, grown as described above, were diluted 1:1,000 in MilliQ water and left to adhere on a poly-l-lysine-coated glass slide (Menzel-Gläser; Thermo Scientific) for 1 h. Cells were washed three times in Dulbecco’s phosphate-buffered saline (DPBS) and fixed with 4% paraformaldehyde at room temperature. Blocking was done using 1.5% BSA, which was followed by washing and cell staining using mAbs at 25 μg/mL for 1 h at room temperature. Alexa Fluor 488 goat anti-mouse IgG antibody (Life Technologies) at a 1:1,000 dilution was added to the slide for 1 h at room temperature, and the slide was washed prior to staining with 25 μg/mL calcofluor white (CFW) to stain cell wall chitin. Mounting medium and a coverslip were added before images were taken using an UltraVIEW VoX spinning-disk confocal microscope (PerkinElmer, Waltham, MA, USA).

### *Ex vivo* macrophage interaction assay. (i) Macrophage culture.

J774.1 mouse macrophage-like cells (ECACC, Salisbury, UK) were cultured in Dulbecco’s modified Eagle medium (DMEM; Thermo Fisher) supplemented with 200 U/mL penicillin/streptomycin, 2 mM l-glutamine (Invitrogen), and 10% (vol/vol) heat-inactivated fetal calf serum in tissue culture flasks at 37°C with 5% CO_2_.

For interaction assays, macrophages were seeded at a density of 1 × 10^5^ cells per well in μ-Slide 8-well chambers (Ibidi) and incubated overnight at 37°C with 5% CO_2_. Immediately before phagocytosis experiments, supplemented DMEM was replaced with prewarmed supplemented CO_2_-independent medium (Thermo Fisher) to ensure that macrophages remained viable during the analysis of C. albicans interactions.

### (ii) C. albicans preincubation with test antibodies.

Prior to phagocytosis assays, C. albicans SC5314 yeast cells (3 × 10^5^ cells) were either untreated or precoated with 50 μg/mL of anti-Pga31, anti-Utr2, or a commercially sourced anti-*Candida* mouse IgG2a monoclonal antibody (catalogue number MA1-7009; Fisher Scientific) in prewarmed supplemented CO_2_-independent medium and incubated at 37°C with gentle shaking for 40 min.

### Live-cell video microscopy of phagocytosis assays.

Video microscopy experiments were performed using an UltraVIEW VoX spinning-disk confocal microscope in a 37°C chamber, with images being captured at 1-min intervals over a 2-h period using a Nikon (Surrey, UK) camera. Six different videos were recorded for each antibody or control group from two biological replicates, and subsequent analysis was conducted using Volocity 6.3 imaging analysis software (PerkinElmer).

At least 25 macrophages were selected at random from each video to determine phagocytic activity. Measurements taken included (i) the time of engulfment, defined as the time between the macrophage establishing cell-cell contact and the complete engulfment of the C. albicans cell, and (ii) the length of intracellular hyphae at two time points (60 and 90 min). Mean values of the lengths of 25 intracellular hyphae for each time point were calculated. A Shapiro-Wilk test for normality was used to determine the distribution of data where appropriate. A Kruskal-Wallis test with Dunn’s multiple-comparison test was used to determine statistical significance using GraphPad Prism 5.

### Investigating the therapeutic efficacy of CWP mAbs in a mouse disseminated candidiasis model.

All animal experimentation was done in accordance with UK Home Office regulations and was approved by both the UK Home Office and an institutional animal welfare and ethical review committee (AWERB). Female BALB/c mice, 7 to 9 weeks old (Envigo Ltd., Huntingdon, UK) were randomly assigned to groups of 6 for treatment and controls. A C. albicans inoculum was prepared by growing strain SC5314 in NGY medium for 16 h with shaking at 30°C. Cells were harvested, washed with saline, counted with a hemocytometer, and resuspended in saline to provide an inoculum of approximately 2.5 × 10^4^ CFU/g mouse body weight in 100 μL. Mice were infected intravenously, and the actual inoculum level was determined by plating cells onto Sabouraud dextrose agar and performing viable cell counts. The range of inoculum levels (calculated based upon the actual body weights of mice) is detailed for each mouse study (see the figure legends). Depending on the study, the treatment dose of mAbs was either 12.5 mg/kg or 15 mg/kg per mouse in 150 μL. mAbs were administered as prophylaxis or treatment. In prophylactic studies, a single dose of antibody was delivered 3 h prior to challenge with the *Candida* inoculum, followed by either single-mAb dosing at 24 h postinfection or double dosing at 24 and 72 h postinfection. For the treatment-only group, two doses of mAb were administered 24 and 72 h after *Candida* challenge. The comparator drug caspofungin was dosed at 1 mg/kg at 24 h and 72 h postinfection. The vehicle-only control followed the same dosing pattern as that for the test mAbs.

Mice were monitored and weighed every day during the course of experiments; at the end of the study period, mice were culled by cervical dislocation; and organs, including kidneys, spleen, and brain, were removed aseptically. Kidneys were split in half, with one half of each kidney used to determine burdens, one half frozen on OCT compound and one half fixed in 10% formalin at 4°C. Tissue sections of kidneys were stained using Gomori’s methenamine silver nitrate staining method. To determine fungal burdens, organ homogenates were plated out in YPD agar and colonies counted after 24 h of growth at 30°C.

In survival studies, mice that lost more than 20% of their initial body weight and/or showed signs of progressive systemic infection were culled by cervical dislocation, and their day of death was recorded as occurring on the following day.

### Statistical analysis.

Statistical analyses of mouse survival data were carried out using IBM SPSS, and GraphPad Prism 5 was used for the rest of the data. For antibody binding curves, data points are expressed as means ± standard errors of the means (SEM) (*n *= 2). For macrophage assays and fungal burdens in mouse kidneys and other organs, results are shown as means ± standard deviations (SD). When comparing two or more groups, a Kruskal-Wallis test with Dunn’s multiple-comparison test was performed to determine statistical significance across all groups and then between different groups when there was a difference across all groups. Mouse survival estimates were compared by the Kaplan-Meier log rank test, with surviving animals being treated as censored data.

### Data availability.

All data are available in the text or the supplemental material. Antibodies described in this paper will be available for research purposes through a material transfer agreement with the University of Aberdeen.
