# Supplementary material for: Monoclonal Antibodies Targeting Surface-Exposed Epitopes of Candida albicans Cell Wall Proteins Confer In Vivo Protection in an Infection Model
Source: Antimicrob Agents Chemother. 2022 Mar 14;66(4):e01957-21. doi: 10.1128/aac.01957-21 (PMC9017365; doi:10.1128/aac.01957-21)
Supplement: Supplemental file 1 — Supplemental material. Download aac.01957-21-s0001.pdf, PDF file, 0.2 MB [file aac.01957-21-s0001.pdf]

Supplementary Materials for

Monoclonal antibodies targeting surface exposed epitopes of *Candida albicans* cell wall proteins confer *in vivo* protection in an infection model

Palliyil et al

Corresponding authors: Soumya Palliyil, soumya.palliyil@abdn.ac.uk, Carol A Munro, c.a.munro@abdn.ac.uk

LIST OF SUPPLEMETARY MATERIALS

Figure S1

Table S1

A

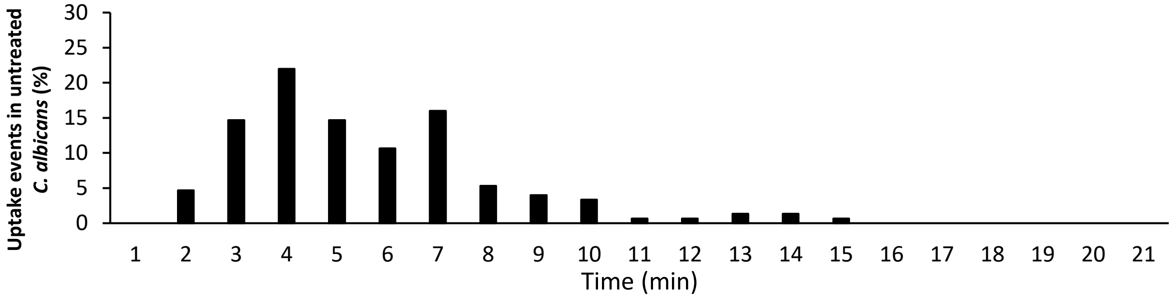

B

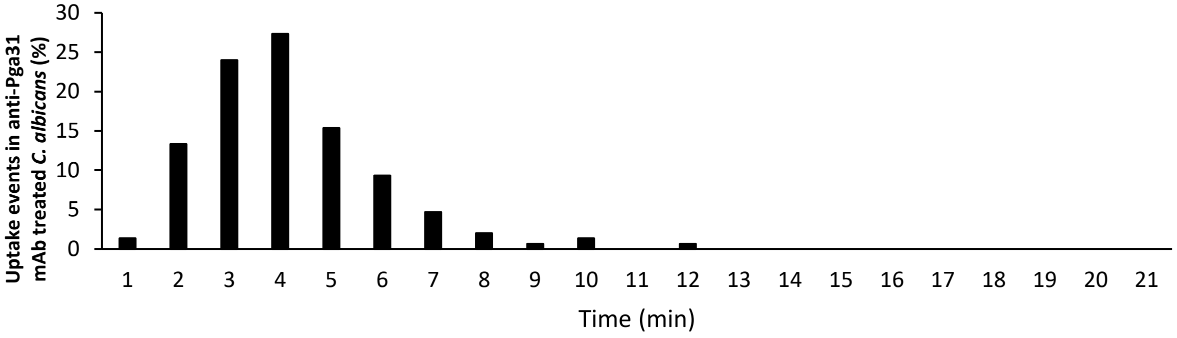

C

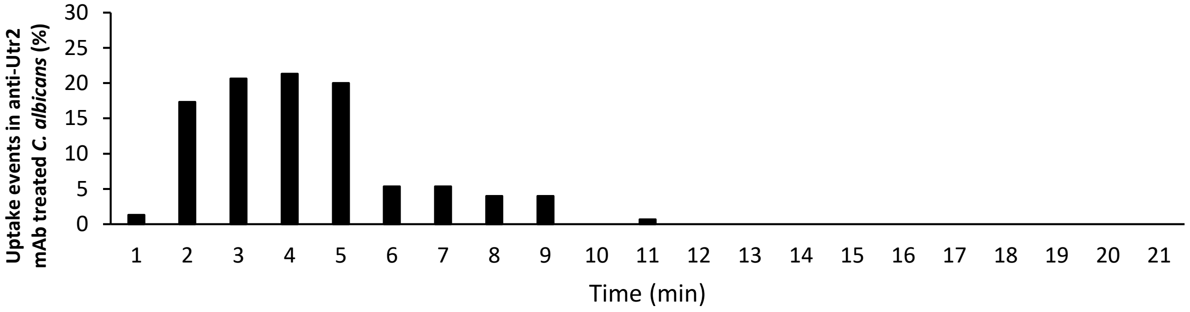

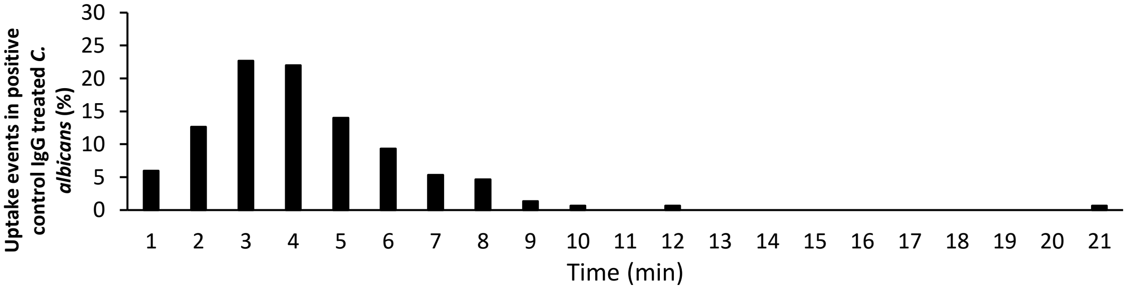

D

Fig. S1. Engulfment of anti-Candida mAb treated* C. albicans *cells by mouse macrophages. *The time taken for J774.1 macrophages to ingest live* Candida *cells following initial cell-cell contact verses the percentage of uptake events are plotted. (A) wt (B) Pga31 mAb (C) Utr2 mAb (D) positive control murine mAb. The rate of engulfment of all antibody-treated cells was faster than that of untreated* C. albicans. *Bars represent the percentages of uptake events (n = 6 videos for each antibody group from two biological replicates).*

Treatment group	Mean (±SD) change in body weight
Isotype Control	-13.77 ± 3.00
Pga31 mAb	-9.49 ± 2.56
Utr2 mAb	-10.79 ± 1.80
Saline only	-11.59 ± 2.89

Table S1. Average weight change in mice in study 1 *Groups of mice (n=6) were treated with either Pga31 mAb (15 mg/kg), Utr2 mAb (15 mg/kg), mouse IgG2a isotype control (15 mg/kg) or saline, 3 h pre and 24 h post-infection in a murine model of disseminated candidiasis. Data represents mean change in body weight ± SD (g) at day 2 compared with day 0 in mouse study 1.*
